# Knowledge, attitudes, and practices of asthma patients in Inner Mongolia regarding differentiation between allergic rhinitis accompanied asthma and cough

**DOI:** 10.3389/fpubh.2025.1624520

**Published:** 2025-12-15

**Authors:** Yihui Wang, Rigai Sa, Hui Ren, Li Du, Boqian Wang, Gang Guo, Jie Tan, Yunfei Bai

**Affiliations:** 1 Inner Mongolia Medical University, Graduate School, Hohhot, China; 2 Department of Respiratory and Critical Care Medicine, The Affiliated Hospital of Inner Mongolia Medical University, Hohhot, China; 3 Department of Otolaryngology Head and Neck Surgery, The Affiliated Hospital of Inner Mongolia Medical University, Hohhot, China

**Keywords:** knowledge, attitude, practice, asthma, allergic rhinitis, coughs

## Abstract

**Objectives:**

This study aimed to evaluate the knowledge, attitudes, and practices (KAP) of asthma patients in Inner Mongolia, focusing on their ability to differentiate between allergic rhinitis accompanied by asthma and common coughs.

**Methods:**

A cross-sectional survey was conducted from Jan 2024 to April 2024 at the Affiliated Hospital of Inner Mongolia Medical University, involving asthma patients aged 18 years and above. Demographic information and KAP scores were gathered through the distribution of questionnaires.

**Results:**

The study successfully collected 547 valid questionnaires. Among the respondents, 310 (56.67%) were female, and 337 (61.61%) either had a personal smoking history or lived with someone who smoked. Median (Q25, Q75) knowledge, attitude, and practice scores were 17 (15, 18) (possible range: 2–18), 43 (41, 44) (possible range: 11–55), and 33 (31, 35) (possible range: 9–45), respectively. Correlation analysis indicated significant positive correlations between knowledge and practice (*r* = 0.2095, *p* < 0.001), as well as attitude and practice (*r* = 0.1420, *p* < 0.001). Pathway results showed that smoking history (*β* = 1.29, *p* < 0.001) directly affected knowledge. Family history (*β* = −0.90, *p* < 0.001) and knowledge (*β* = 0.15, *p* < 0.001) directly affected attitude. Knowledge (*β* = 0.43, *p* < 0.001) and attitude (*β* = 0.30, *p* < 0.001) directly affected practice. Indirect effects analyses also showed that smoking history (*β* = 0.19, *p* = 0.002) had an indirect effect on attitude. Smoking history (*β* = 0.47, *p* < 0.001), family history (*β* = −0.57, *p* < 0.001), and knowledge (*β* = 0.06, *p* < 0.001) had indirect effects on practice.

**Conclusion:**

Asthma patients in Inner Mongolia demonstrated moderate knowledge, attitudes, and proactive practices toward distinguishing between allergic rhinitis accompanied by asthma and common coughs. Based on these findings, we recommend strengthening targeted patient education in clinical practice, particularly for individuals with lower knowledge levels, a smoking history, or limited awareness of family health history, to enhance symptom recognition and self-management capacity.

## Background

Cough, with a prevalence reaching 33% of the population, is the most common symptom prompting individuals to seek medical advice (1). This symptom can be indicative of various respiratory and non-respiratory diseases, such as chronic rhinosinusitis, asthma, Chronic Obstructive Pulmonary Disease (COPD), pneumonia, and chronic bronchitis (1, 2). Among these conditions, cough variant asthma (CVA) represents a unique subtype of asthma characterized solely by chronic cough, lacking other common asthma symptoms like wheezing, chest tightness, or dyspnea. This often leads to its misdiagnosis or complete non-diagnosis (3).

In China, asthma affects approximately 4.2% of adults, translating to an estimated 45.7 million individuals living with the condition (4). A specific study in the grassland regions of Northern China highlights that the prevalence rates of allergic rhinitis (AR), asthma, and concurrent AR and asthma are 13.9, 9.8, and 2.9%, respectively (5). The diversity and abundance of grass pollen in these vegetative areas contribute to the high incidence of these conditions, making such regions critical for the prevention and treatment of AR and asthma (6).

The Knowledge-Attitude-Practice (KAP) theory, which plays a pivotal role in shaping human health behaviors, is often utilized to comprehensively assess the knowledge, attitudes, and practices of a target population within the healthcare sector (7). This model, fundamental to health literacy, posits that knowledge positively influences attitudes, which in turn shape individual practices (8). Employing the KAP questionnaire facilitates the evaluation of both the demand and level of acceptance of relevant content, providing a detailed understanding of the target group’s health behaviors (9). The clinical challenge in regions like Inner Mongolia involves distinguishing between allergic rhinitis accompanied by asthma and cough variant asthma, exacerbated by diverse grass pollens that significantly impact respiratory symptoms. Such differentiation is critical, as it influences the accuracy of diagnosis and the effectiveness of subsequent treatment strategies. No previous KAP studies have targeted this specific aspect in Inner Mongolia, underscoring the necessity of this research.

This study, therefore, aims to evaluate the KAP of asthma patients in Inner Mongolia, with a specific focus on their ability to differentiate between allergic rhinitis accompanied by asthma and common coughs. By enhancing diagnostic precision and patient education, the research seeks to develop tailored interventions that effectively address the overlapping symptoms and prevent clinical oversight of these conditions in a high-risk area.

## Methods

### Study design and participants

This cross-sectional study was conducted from Jan 2024 to April 2024 at the Affiliated Hospital of Inner Mongolia Medical University, focusing on patients with asthma. The study was approved by the Ethics Committee of the author’s Hospital, and informed consent was obtained from all participants.

Inclusion criteria: Patients clinically diagnosed with asthma at the Affiliated Hospital of Inner Mongolia Medical University, aged 18 years or older.

Exclusion criteria: Patients suffering from other systemic diseases such as autoimmune disorders, malignant tumors, or conditions not related to asthma were excluded from the study.

To collect data, convenience sampling was employed to distribute questionnaires to both outpatients and inpatients. Participants were invited to complete the questionnaire by scanning a QR code with their mobile phones on-site. For discharged patients, the QR code was distributed through WeChat groups to facilitate remote participation. All data collection was conducted by trained research personnel at the outpatient and inpatient departments of the Department of Respiratory and Critical Care Medicine. During the survey, some older participants or those with low literacy had difficulty operating smartphones or understanding the questions. To address this issue, our trained staff provided one-on-one assistance, including reading the questions aloud and helping operate the questionnaire system based on the participant’s verbal responses. This ensured accurate and complete data recording.

### Sample size calculation

The required sample size was estimated using the formula for cross-sectional studies (10):

*n* = *Z*^2^ × *p*(1 − *p*)/*d*^2^,

where *n* is the sample size, *Z* is the *Z*-score corresponding to a 95% confidence level (1.96), *p* is the expected proportion (set conservatively at 0.5 to maximize sample size), and d is the margin of error (set at 0.05). Based on this, the minimum required sample size was *n* = (1.96^2^ × 0.5 × 0.5)/0.05^2^ = 384.16, rounded up to 385.

A total of 547 valid questionnaires were collected, which exceeds the minimum requirement and ensures adequate statistical power for the study analyses.

### Procedures

The questionnaire design underwent modification after consultation with three experts from the fields of respiratory medicine and otorhinolaryngology. Following their feedback, a pilot study was executed involving 50 randomly selected asthma patients, which yielded a high reliability coefficient of 0.9653. Any issues identified during the pilot phase were quickly resolved to enhance the survey’s effectiveness.

The refined questionnaire encompassed four dimensions, presented in Chinese. These included demographic information with 12 questions; a knowledge dimension consisting of 13 questions, with answers scored as 2 points for a correct response, 1 point for recognition, and 0 points for uncertainty, including some multiple-choice questions that allowed a scoring range from 2 to 18 points; an attitude dimension with 11 questions rated on a 5-point Likert scale, where the intensity of the attitude corresponded to scores ranging from 1 to 5, yielding a total possible score between 11 and 55 points; and a practice dimension comprising 9 questions, also evaluated on a 5-point Likert scale, scoring actions from 1 to 5, with total scores ranging from 9 to 45 points. Scores exceeding 70% of the maximum possible in each section were indicative of adequate knowledge, a positive attitude, and proactive practice (11).

During the formal survey phase, the questionnaire was integrated into the Questionnaire Star platform and disseminated via QR codes. Survey administrators were responsible for promptly verifying the completeness and accuracy of the responses upon submission. To minimize data entry errors and ensure data integrity, all responses were double-entered and each questionnaire was assigned a unique identifier. The research team meticulously reviewed the completeness, internal consistency, and logical coherence of each questionnaire, ensuring the reliability of the collected data.

### Statistical analyses

Data analysis was conducted using R 4.3.2. Continuous data are presented as means and standard deviations (SD) for normally distributed variables, and medians along with the 25th and 75th percentiles for non-normally distributed variables. Categorical data are expressed as counts and percentages [*N*(%)], describing responses across different demographic characteristics. For continuous variables, a normality test was applied. Comparisons between two groups for normally distributed variables were made using the independent samples *t*-test, while the Wilcoxon Mann–Whitney test was utilized for non-normally distributed data. For comparisons among three or more groups, ANOVA was conducted for variables with normal distribution and uniform variance, and the Kruskal–Wallis test was employed for non-normally distributed data. Correlation analysis was performed using the Pearson correlation coefficient for normally distributed data and the Spearman correlation coefficient for non-normally distributed data. Regression analyses, both univariate and multivariate, were carried out with dimension scores as dependent variables to investigate the relationships between demographic data and scores. Variables that achieved a *p*-value of less than 0.1 in univariate analyses were included in the multivariate regression. Classification of mean scores was used for normally distributed data, whereas median scores were used for non-normally distributed data. *p*-values were reported to three decimal places, considering *p* < 0.05 as statistically significant. Following the theoretical framework of Knowledge, Attitudes, and Practices (KAP), structural equation modeling (SEM) was used to examine whether attitudes act as mediators between knowledge and practices. Direct and indirect effects were computed and compared. Where model fit thresholds were not met, path analysis was employed for mediation testing. Statistical analyses involving SEM were performed using Stata 18.0.

## Results

A total of 564 participants were initially recruited for the study. Exclusions were made as follows: nine participants under the age of 18 (Question 2: “Your age”), one participant with incomplete data on the duration of asthma diagnosis (Question 8: “How long have you been diagnosed with asthma?”), one participant each for incomplete responses to questions 3 and 4 in the knowledge section, one participant for an incomplete response to question 5, three participants for incomplete responses to question 8 in the attitude section, and one participant for an incomplete response to question 9 in the practice section. Consequently, the final dataset comprised 547 participants, resulting in an effectiveness rate of 96.99%. The overall Cronbach’s *α* coefficient for the validated survey questionnaire was 0.7174, demonstrating good internal consistency. The Kaiser–Meyer–Olkin (KMO) measure was 0.690 (*p* < 0.001).

### Demographic characteristics and KAP scores

The study included 547 participants with an average age of 42.66 ± 12.35 years. Of these, 310 (56.67%) were female, 195 (35.65%) had attained a college or bachelor’s degree, 354 (64.72%) resided in urban areas, 299 (54.66%) frequently experienced coughing, 251 (45.89%) had been diagnosed with asthma for no more than 3 years, and 388 (70.93%) had a history of allergic rhinitis. Additionally, 337 (61.61%) of the participants or their primary cohabitants had a history of smoking. Among the 547 participants, the knowledge scores ranged from 2 to 18, with a median of 17, and 63.6% scored above the 70% adequacy threshold. The attitude scores ranged from 11 to 55, with a median of 43, and 72.3% of participants scored above 38.5. The practice scores ranged from 9 to 45, with a median of 33, and 58.4% exceeded the 70% cutoff of 31.5. These results suggest that while general awareness and attitudes are relatively strong, there is more variability in practice behaviors. Analysis of demographic characteristics revealed that knowledge scores varied significantly based on gender (*p* = 0.003), education level (*p* < 0.001), occupation (*p* < 0.001), place of residence (*p* = 0.014), the presence of immediate family members with asthma (*p* = 0.030), a history of allergic rhinitis (*p* = 0.008), social medical insurance status (*p* < 0.001), and smoking history (*p* < 0.001). Having an immediate family member with asthma also significantly influenced attitudes (*p* = 0.002), and educational level, allergic rhinitis history, and smoking history were predictors of varying practice scores (*p* = 0.040, *p* = 0.008, and *p* = 0.044, respectively) (Table 1).

**Table 1:** Demographic characteristics of participants and their corresponding KAP scores.

*N* = 547	*N* (%)	Knowledge score		Attitude score		Practice score	
		Med(q25,q75)	*P*	Med(q25,q75)	*P*	Med(q25,q75)	*P*
Total Score		17(15,18)		43(41,44)		33(31,35)	
Gender			**0.003**		0.164		0.888
a. Male	237(43.33)	17(15,18)		43(41,44)		33(30,35)	
b. Female	310(56.67)	17(16,19)		43(41,44)		33(31,35)	
Age (years old)[Range:18 ~ 77]	42.66 ± 12.35						
Education			**<0.001**		0.328		**0.040**
a. Middle school and below	121(22.12)	16(14,18)		43(41,44)		33(31,35)	
b. High school/ technical secondary school	111(20.29)	16(14,18)		43(41,44)		32(30,35)	
c. College / Bachelor’s degree	195(35.65)	17(15,18)		43(41,45)		33(30,35)	
d. Master’s degree and above	120(21.94)	18(17,19.5)		43(41,45)		34(31.5,36)	
Occupation			**<0.001**		0.300		0.180
a. Medical personnel	11(2.01)	20(18,22)		44(42,48)		34(32,36)	
b. Farmer	75(13.71)	16(14,18)		43(40,44)		33(31,35)	
c. Enterprise employee	131(23.95)	17(16,19)		43(42,44)		33(31,35)	
d. Public servant (government/institution staff, etc.)	85(15.54)	18(16,19)		43(41,45)		34(31,36)	
e. Worker (e.g., construction industry)	22(4.02)	16.5(14,18)		42.5(41,44)		31.5(27,36)	
f. Freelancer	72(13.16)	16(15,17.5)		42(41,44)		32(30,34)	
g. Self-employed / Businessperson	60(10.97)	16(14,18)		43(41,44)		33(30,35)	
h. Retired	48(8.78)	17(15,18)		43(41.5,44)		33(31,35)	
i. Other	43(7.86)	17(16,20)		43(42,45)		34(31,37)	
Residence			**0.014**		0.389		0.123
a. Urban	354(64.72)	17(15,19)		43(41,44)		33(30,36)	
b. Suburban/rural	193(35.28)	17(15,18)		43(41,44)		33(31,35)	
In the past year, your family’s monthly per capita income was (including in-kind income and rental income, etc.):______			0.332		0.593		0.440
a. Below 2000	79(14.44)	16(14,18)		43(40,45)		33(31,35)	
b. 2001–5,000	185(33.82)	17(15,19)		43(41,44)		33(31,35)	
c. 5001–10,000	156(28.52)	17(16,18.5)		43(41,45)		33(30.5,35.5)	
d. 10001–20,000	92(16.82)	17(15,18)		43(41,45)		32(29.5,34.5)	
e. Above 20,000	35(6.4)	16(15,18)		42(40,44)		33(30,35)	
Do you frequently cough:			0.398		0.565		0.390
a. Yes	299(54.66)	17(15,18)		43(41,44)		33(31,35)	
b. No	248(45.34)	17(15,19)		43(41,44.5)		33(30,35)	
How long have you been diagnosed with asthma:			0.123		0.085		0.276
a. ≦3 years	251(45.89)	17(15,18)		43(41,45)		33(31,35)	
b. 3–5 years	119(21.76)	17(15,19)		43(41,45)		33(31,36)	
c. 5–10 years	125(22.85)	17(15,18)		42(41,44)		32(30,35)	
d. ≧10 years	52(9.51)	18(16,19.5)		43(41.5,44)		33(30,35)	
Do you have any immediate family members with asthma (blood relatives):			**0.030**		**0.002**		0.113
a. Yes	181(33.09)	17(15,19)		43(42,45)		33(31,36)	
b. No	366(66.91)	17(15,18)		43(41,44)		33(31,35)	
Do you have a history of allergic rhinitis, such as pollen, pets, dust mites, etc.:			**0.008**		0.637		**0.008**
a. Yes	388(70.93)	17(15,19)		43(41,44)		33(31,35)	
b. No	159(29.07)	16(15,18)		43(41,44)		32(30,35)	
Are you participating in a social medical insurance plan:			**<0.001**		0.213		0.902
a. Yes	534(97.62)	17(15,18)		43(41,44)		33(31,35)	
b. No	13(2.38)	12(10,15)		45(40,46)		32(31,36)	
Do you or your main cohabitant have a history of smoking:			**<0.001**		0.186		**0.044**
a. Yes	337(61.61)	16(15,18)		43(41,44)		33(31,35)	
b. No	210(38.39)	17.5(16,19)		43(41,44)		33(30,36)	

Bolded *P*-values indicate statistically significant differences (*P* < 0.05).

### The overall scores for the knowledge, attitude and practice sections, respectively

The distribution of knowledge dimensions shown that the three questions with the highest number of participants choosing the “Not clear” option were “The incidence of asthma in allergic rhinitis patients is 4–20 times higher than that in normal individuals. The incidence of asthma in the general population is about 2–5%, while in allergic rhinitis patients, it can be as high as 20–40%.” (K3) with 45.52%, “Allergic rhinitis accompanied by asthma is part of the body’s immune response to recognized foreign substances.” (K7) with 44.%, and “A family history of allergies is a major risk factor for allergic asthma.” (K8) with 24.31% (Table 2).

**Table 2:** Distribution of participant responses for each item in the knowledge dimension.

Knowledge	*N*(%)		
	Very familiar	Heard of it	Not clear
1. Allergic rhinitis (allergic rhinitis) refers to chronic allergic inflammation of the nasal mucosa, commonly known as “upper respiratory tract sensitivity.” Typical symptoms include recurrent watery nasal discharge, sneezing, nasal congestion, and itching of the nose/eyes.	79(14.44)	348(63.62)	120(21.94)
2. Allergic rhinitis can trigger allergic asthma, urticaria, and other complications.	174(31.81)	323(59.05)	50(9.14)
3. The incidence of asthma in allergic rhinitis patients is 4–20 times higher than that in normal individuals. The incidence of asthma in the general population is about 2–5%, while in allergic rhinitis patients, it can be as high as 20–40%.	49(8.96)	249(45.52)	249(45.52)
4. Allergic rhinitis accompanied by asthma often occurs in which seasons?			
a. Spring	189(34.55)		
b. Summer	159(29.07)		
c. Autumn	154(28.15)		
d. Winter	45(8.23)		
5. Coughing often occurs in which seasons?			
a. Spring	50(9.14)		
b. Summer	/		
c. Autumn	258(47.17)		
d. Winter	239(43.69)		
6. The symptoms of allergic rhinitis typically include sneezing, runny nose, and conjunctivitis; asthma, besides evident lung function changes, also presents symptoms such as wheezing, coughing, and shortness of breath.	100(18.28)	363(66.36)	84(15.36)
7. Allergic rhinitis accompanied by asthma is part of the body’s immune response to recognized foreign substances.	35(6.4)	271(49.54)	241(44.06)
8. A family history of allergies is a major risk factor for allergic asthma.	103(18.83)	311(56.86)	133(24.31)
9. Rhinitis is an independent risk factor for recurrent coughing and wheezing in childhood.	196(35.83)	253(46.25)	98(17.92)
10. Coughing is a reflex action to clear mucus and irritants (e.g., dust or smoke) from the airways.	303(55.39)	224(40.95)	20(3.66)
11. Asthma is a common chronic condition that can lead to coughing, wheezing, chest tightness, and difficulty breathing.	350(63.99)	186(34)	11(2.01)
12. The treatment of cough mainly includes the following options (multiple choices):			
a. Antitussive drugs	543(99.27)		
b. Expectorants	544(99.45)		
c. Local treatment (emotional soothing effect)	505(92.32)		
d. Cough promoters	471(86.11)		
e. Bronchodilators	521(95.25)		
13. Treatment options for allergic rhinitis and asthma include the following (multiple choices):			
a. Glucocorticoid therapy	541(98.9)		
b. Antiallergic drugs	541(98.9)		
c. Allergen immunotherapy	503(91.96)		
d. Anti-IgE monoclonal antibody	503(91.96)		
e. Symptomatic treatment	525(95.98)		

Values are presented as frequencies and percentages.

When it comes to related attitudes, over 64% of participants (n = 351) disagreed or strongly disagreed with the statement “I have enough knowledge to differentiate between allergic rhinitis accompanied by asthma and normal cough” (A9), indicating a significant lack of confidence in symptom distinction. In contrast, 79.2% (*n* = 434) either agreed or strongly agreed that it is important to understand the difference between asthma and allergic rhinitis for disease management (A2). Furthermore, 92.3% (*n* = 506) expressed willingness to actively learn how to differentiate between the conditions (A10), suggesting a high potential for educational interventions despite current knowledge gaps (Table 3).

**Table 3:** Distribution of participant responses for each item in the attitude dimension.

Attitude	Strongly agree	Agree	Neutral	Disagree	Strongly disagree
1. Do you believe that allergic rhinitis accompanied by asthma is completely different from simple coughing and should actively seek medical help for appropriate treatment? (P)	86(15.72)	410(74.95)	51(9.32)		
2. Do you think understanding the differences between asthma and allergic rhinitis is very important for disease management? (P)	126(23.03)	295(53.93)	118(21.57)	8(1.46)	
3. Are you willing to actively seek medical help to differentiate the symptoms of asthma, allergic rhinitis, and coughing? (P)	75(13.71)	376(68.74)	94(17.18)	2(0.37)	
4. Do you believe that effectively managing allergic rhinitis and asthma will improve your quality of life? (P)	175(31.99)	341(62.34)	30(5.48)	1(0.18)	
5. Do you support treatment recommendations for allergic rhinitis accompanied by asthma from doctors or medical institutions? (P)	147(26.87)	356(65.08)	43(7.86)	1(0.18)	
6. Do you think the symptoms of asthma and coughing could affect your work or daily life? (P)	109(19.93)	428(78.24)	10(1.83)		
7. Regarding the treatment of allergic rhinitis and asthma, are you open-minded and willing to try new methods or medications? (P)	104(19.01)	261(47.71)	145(26.51)	37(6.76)	
8. Do you think being able to accurately distinguish between allergic rhinitis accompanied by asthma and normal coughing is crucial for your health management?	58(10.6)	369(67.46)	115(21.02)	5(0.91)	
9. Do you feel you have enough knowledge to differentiate between allergic rhinitis accompanied by asthma and normal coughing?	18(3.29)	18(3.29)	160(29.25)	277(50.64)	74(13.53)
10. Are you willing to actively learn how to differentiate between allergic rhinitis accompanied by asthma and coughing?	128(23.4)	372(68.01)	47(8.59)		
11. Do you think improving your ability to differentiate between allergic rhinitis accompanied by asthma and coughing will help reduce unnecessary treatment and medical costs?	209(38.21)	242(44.24)	96(17.55)		

Values are presented as frequencies and percentages.

In terms of practice behaviors, 27.61% (*n* = 151) rarely used different medications to treat allergic rhinitis accompanied by asthma and cough separately (P4), while 38.21% (*n* = 209) only “sometimes” did so. Similarly, only 5.3% (*n* = 29) reported always educating their family members and friends about asthma and cough (P9), while 22.12% (*n* = 121) reported rarely doing so. A total of 18.46% (*n* = 101) reported “rarely” or “never” recording asthma or cough symptoms for better disease management (P5), suggesting a need to reinforce symptom tracking and patient engagement strategies (Table 4).

**Table 4:** Distribution of participant responses for each item in the practice dimension.

Practice	Always	Often	Sometimes	Rarely	Never
1. Do you actively seek medical help to determine whether the disease is allergic rhinitis accompanied by asthma or normal coughing? (P)	98(17.92)	219(40.04)	161(29.43)	69(12.61)	/
2. Do you take proactive measures to reduce exposure to allergens to lower the risk of asthma attacks? (P)	183(33.46)	292(53.38)	64(11.7)	8(1.46)	/
3. Do you regularly use medication to control symptoms of allergic rhinitis accompanied by asthma? (P)	171(31.26)	120(21.94)	179(32.72)	77(14.08)	/
4. Do you use different medications to treat allergic rhinitis accompanied by asthma and coughing? (P)	20(3.66)	167(30.53)	209(38.21)	151(27.61)	/
5. Do you consciously keep records of asthma and coughing symptoms for better disease management? (P)	37(6.76)	93(17)	316(57.77)	57(10.42)	44(8.04)
6. Do you actively restrict smoking to improve asthma symptoms? (P)	310(56.67)	139(25.41)	70(12.8)	18(3.29)	10(1.83)
7. Do you actively participate in medical examinations to monitor allergic rhinitis accompanied by asthma? (P)	158(28.88)	103(18.83)	200(36.56)	81(14.81)	5(0.91)
8. Do you take proactive measures to improve home and work environments to reduce allergen exposure? (P)	233(42.6)	135(24.68)	116(21.21)	52(9.51)	11(2.01)
9. Do you take proactive measures to educate your family members and friends to help them better understand asthma and coughing? (P)	29(5.3)	224(40.95)	166(30.35)	121(22.12)	7(1.28)

Values are presented as frequencies and percentages.

### Correlations between knowledge, attitude, and practice scores

Correlation analysis demonstrated that both knowledge and attitude scores were significantly associated with practice. Specifically, knowledge and practice showed a moderate positive correlation (*r* = 0.2095, *p* < 0.001), while attitude and practice had a weaker but still significant correlation (*r* = 0.1420, *p* < 0.001). No statistically significant correlation was observed between knowledge and attitude (*r* = 0.0804, *p* = 0.0601), indicating that knowledge alone may not directly influence attitudes without other mediating factors (Table 5).

**Table 5:** Spearman correlation coefficients between knowledge, attitude, and practice scores.

Variables	Knowledge	Attitude	Practice
Knowledge	1		
Attitude	0.0804 (*P* = 0.0601)	1	
Practice	0.2095 (*P* < **0.001**)	0.1420 (*P* < **0.001**)	1

Bolded *p*-values indicate statistically significant differences (*P* < 0.05).

### Factor analysis related to knowledge, attitude, and practice

Multivariate logistic regression showed that being farmer (OR = 0.09, 95% CI: [0.01,0.87], *p* = 0.038), being freelancer (OR = 0.06, 95% CI: [0.00,0.59], *p* = 0.015), being self-employed / businessperson (OR = 0.10, 95% CI: [0.01,0.95], *p* = 0.045), other occupation (OR = 0.10, 95% CI: [0.01,0.98], *p* = 0.049), without a family history of asthma (OR = 0.65, 95% CI: [0.44,0.96], *p* = 0.032), and without history of smoking (OR = 1.67, 95% CI: [1.15,2.43], *p* = 0.007) were independently associated with poor knowledge. Meanwhile, diagnosed with asthma for 5–10 years (OR = 0.59, 95% CI: [0.37,0.93], *p* = 0.024) and without a family history of asthma (OR = 0.62, 95% CI: [0.43,0.89], *p* = 0.011) were independently associated with negative attitude. Furthermore, knowledge score (OR = 1.12, 95% CI: [1.05,1.19], *p* < 0.001), attitude score (OR = 1.07, 95% CI: [1.00,1.14], *p* = 0.027), being male (OR = 0.357, 95% CI: [0.136–0.943], *p* = 0.038) were independently associated with positive practice (Table 6).

**Table 6:** Univariate and multivariate linear regression analysis of demographic predictors of knowledge scores.

Knowledge	Univariate analysis		Multivariate analysis	
	OR (95%CI)	*P*	OR (95%CI)	*P*
Gender				
a. Male				
b. Female	1.38(0.97,1.96)	0.066		
Age (years old)[Range: 18 ~ 77]	0.98(0.97,1.00)	0.057		
Shared decision-making [Range: 18 ~ 54]	1.01(0.98,1.03)	0.272		
Education				
a. Middle school and below				
b. High school/technical secondary school	1.04(0.60,1.82)	0.867	0.93(0.51,1.69)	0.818
c. College / Bachelor’s degree	1.39(0.86,2.25)	0.174	1.15(0.64,2.07)	0.624
d. Master’s degree and above	2.85(1.68,4.83)	**0**	1.81(0.93,3.51)	0.077
Occupation				
a. Medical personnel				
b. Farmer	0.04(0.00,0.34)	**0.003**	0.09(0.01,0.87)	**0.038**
c. Enterprise employee	0.08(0.01,0.67)	**0.02**	0.13(0.01,1.16)	0.069
d. Public servant (government/institution staff, etc.)	0.10(0.01,0.83)	**0.033**	0.16(0.01,1.38)	0.097
e. Worker (e.g., construction industry)	0.05(0.00,0.53)	**0.012**	0.12(0.01,1.26)	0.079
f. Freelancer	0.03(0.00,0.27)	**0.002**	0.06(0.00,0.59)	**0.015**
g. Self-employed/Businessperson	0.05(0.00,0.44)	**0.007**	0.10(0.01,0.95)	**0.045**
h. Retired	0.07(0.00,0.60)	**0.015**	0.15(0.01,1.43)	0.101
i. Other	0.05(0.00,0.50)	**0.01**	0.10(0.01,0.98)	**0.049**
Residence				
a. Urban				
b. Suburban/rural	0.71(0.49,1.02)	0.07		
In the past year, your family’s monthly per capita income was (including in-kind income and rental income, etc.):______				
a. Below 2000				
b. 2001–5,000	1.55(0.89,2.69)	0.118		
c. 5001–10,000	1.34(0.76,2.37)	0.307		
d. 10001–20,000	1.37(0.73,2.56)	0.324		
e. Above 20,000	1.20(0.52,2.76)	0.661		
Do you frequently cough:				
a. Yes				
b. No	0.91(0.64,1.29)	0.619		
How long have you been diagnosed with asthma:				
a. ≦3 years				
b. 3–5 years	1.18(0.76,1.85)	0.443		
c. 5–10 years	0.95(0.61,1.49)	0.843		
d. ≧10 years	1.77(0.97,3.23)	0.062		
Do you have any immediate family members with asthma (blood relatives):				
a. Yes				
b. No	0.60(0.41,0.86)	**0.006**	0.65(0.44,0.96)	**0.032**
Do you have a history of allergic rhinitis, such as pollen, pets, dust mites, etc.:				
a. Yes				
b. No	0.62(0.42,0.92)	**0.018**	0.76(0.50,1.16)	0.207
Are you participating in a social medical insurance plan:				
a. Yes				
b. No	0.26(0.05,1.21)	0.089		
Do you or your main cohabitant have a history of smoking:				
a. Yes				
b. No	1.98(1.39,2.82)	**<0.001**	1.67(1.15,2.43)	**0.007**

Attitude	Univariate analysis		Multivariate analysis	
	OR (95%CI)	*P*	OR (95%CI)	*P*
Knowledge score	1.01(0.96,1.07)	0.497		
Gender				
a. Male				
b. Female	1.13(0.80,1.59)	0.477		
Age (years old)[Range: 18 ~ 77]	0.99(0.98,1.01)	0.95		
Shared decision-making [Range: 18 ~ 54]	0.97(0.95,1.00)	0.088		
Education				
a. Middle school and below				
b. High school/ technical secondary school	1.24(0.73,2.10)	0.418		
c. College / Bachelor’s degree	1.23(0.77,1.96)	0.374		
d. Master’s degree and above	1.01(0.60,1.70)	0.96		
Occupation				
a. Medical personnel				
b. Farmer	0.62(0.17,2.21)	0.462		
c. Enterprise employee	0.58(0.16,2.01)	0.395		
d. Public servant (government/institution staff, etc.)	0.50(0.14,1.78)	0.287		
e. Worker (e.g., construction industry)	0.47(0.10,2.07)	0.323		
f. Freelancer	0.63(0.17,2.25)	0.478		
g. Self-employed / Businessperson	0.41(0.11,1.53)	0.188		
h. Retired	0.59(0.15,2.22)	0.441		
i. Other	0.79(0.21,3.00)	0.736		
Residence				
a. Urban				
b. Suburban/rural	1.03(0.72,1.47)	0.861		
In the past year, your family’s monthly per capita income was (including in-kind income and rental income, etc.):______				
a. Below 2000				
b. 2001–5,000	0.90(0.53,1.55)	0.726		
c. 5001–10,000	1.10(0.63,1.91)	0.719		
d. 10001–20,000	0.93(0.50,1.72)	0.837		
e. Above 20,000	0.72(0.31,1.66)	0.451		
Do you frequently cough:				
a. Yes				
b. No	1.01(0.71,1.42)	0.938		
How long have you been diagnosed with asthma:				
a. ≦3 years				
b. 3–5 years	1.13(0.73,1.76)	0.564	1.10(0.71,1.72)	0.649
c. 5–10 years	0.60(0.38,0.94)	**0.027**	0.59(0.37,0.93)	**0.024**
d. ≧10 years	0.97(0.53,1.77)	0.924	0.95(0.51,1.75)	0.88
Do you have any immediate family members with asthma (blood relatives):				
a. Yes				
b. No	0.62(0.43,0.89)	**0.011**	0.62(0.43,0.89)	**0.011**
Do you have a history of allergic rhinitis, such as pollen, pets, dust mites, etc.:				
a. Yes				
b. No	1.03(0.71,1.50)	0.865		
Are you participating in a social medical insurance plan:				
a. Yes				
b. No	2.35(0.76,7.29)	0.137		
Do you or your main cohabitant have a history of smoking:				
a. Yes				
b. No	1.00(0.70,1.41)	0.999		

Practice	Univariate analysis		Multivariate analysis	
	OR (95%CI)	*P*	OR (95%CI)	*P*
Knowledge score	1.14(1.07,1.21)	**<0.001**	1.12(1.05,1.19)	**<0.001**
Attitude score	1.09(1.02,1.16)	**0.004**	1.07(1.00,1.14)	**0.027**
Gender				
a. Male				
b. Female	0.97(0.69,1.36)	0.878		
Age (years old)[Range: 18 ~ 77]	0.98(0.97,1.00)	0.071		
Shared decision-making [Range: 18 ~ 54]	1.03(1.00,1.05)	**0.016**	1.02(0.99,1.05)	0.077
Education				
a. Middle school and below				
b. High school/technical secondary school	0.77(0.45,1.31)	0.349		
c. College/Bachelor’s degree	0.94(0.59,1.49)	0.801		
d. Master’s degree and above	1.51(0.91,2.52)	0.108		
Occupation				
a. Medical personnel				
b. Farmer	0.40(0.10,1.49)	0.174		
c. Enterprise employee	0.41(0.11,1.48)	0.175		
d. Public servant (government/institution staff, etc.)	0.61(0.16,2.25)	0.461		
e. Worker (e.g., construction industry)	0.26(0.05,1.22)	0.089		
f. Freelancer	0.30(0.08,1.13)	0.077		
g. Self-employed/Businessperson	0.40(0.10,1.54)	0.187		
h. Retired	0.48(0.12,1.87)	0.293		
i. Other	0.59(0.15,2.34)	0.462		
Residence				
a. Urban				
b. Suburban/rural	0.87(0.61,1.25)	0.473		
In the past year, your family’s monthly per capita income was (including in-kind income and rental income, etc.):______				
a. Below 2000				
b. 2001–5,000	1.59(0.93,2.74)	0.089		
c. 5001–10,000	1.51(0.87,2.64)	0.142		
d. 10001–20,000	1.01(0.54,1.88)	0.973		
e. Above 20,000	1.01(0.44,2.32)	0.965		
Do you frequently cough:				
a. Yes				
b. No	0.94(0.67,1.32)	0.741		
How long have you been diagnosed with asthma:				
a. ≦3 years				
b. 3–5 years	1.00(0.64,1.54)	1		
c. 5–10 years	0.72(0.46,1.12)	0.151		
d. ≧10 years	0.67(0.36,1.24)	0.203		
Do you have any immediate family members with asthma (blood relatives):				
a. Yes				
b. No	0.75(0.53,1.08)	0.131		
Do you have a history of allergic rhinitis, such as pollen, pets, dust mites, etc.:				
a. Yes				
b. No	0.68(0.47,1.00)	0.054		
Are you participating in a social medical insurance plan:				
a. Yes				
b. No	0.80(0.26,2.50)	0.711		
Do you or your main cohabitant have a history of smoking:				
a. Yes				
b. No	1.39(0.98,1.97)	0.06		

Bolded *P*-values indicate statistically significant differences (*P* < 0.05).

### The structural equation model (SEM)

**Figure 1:**
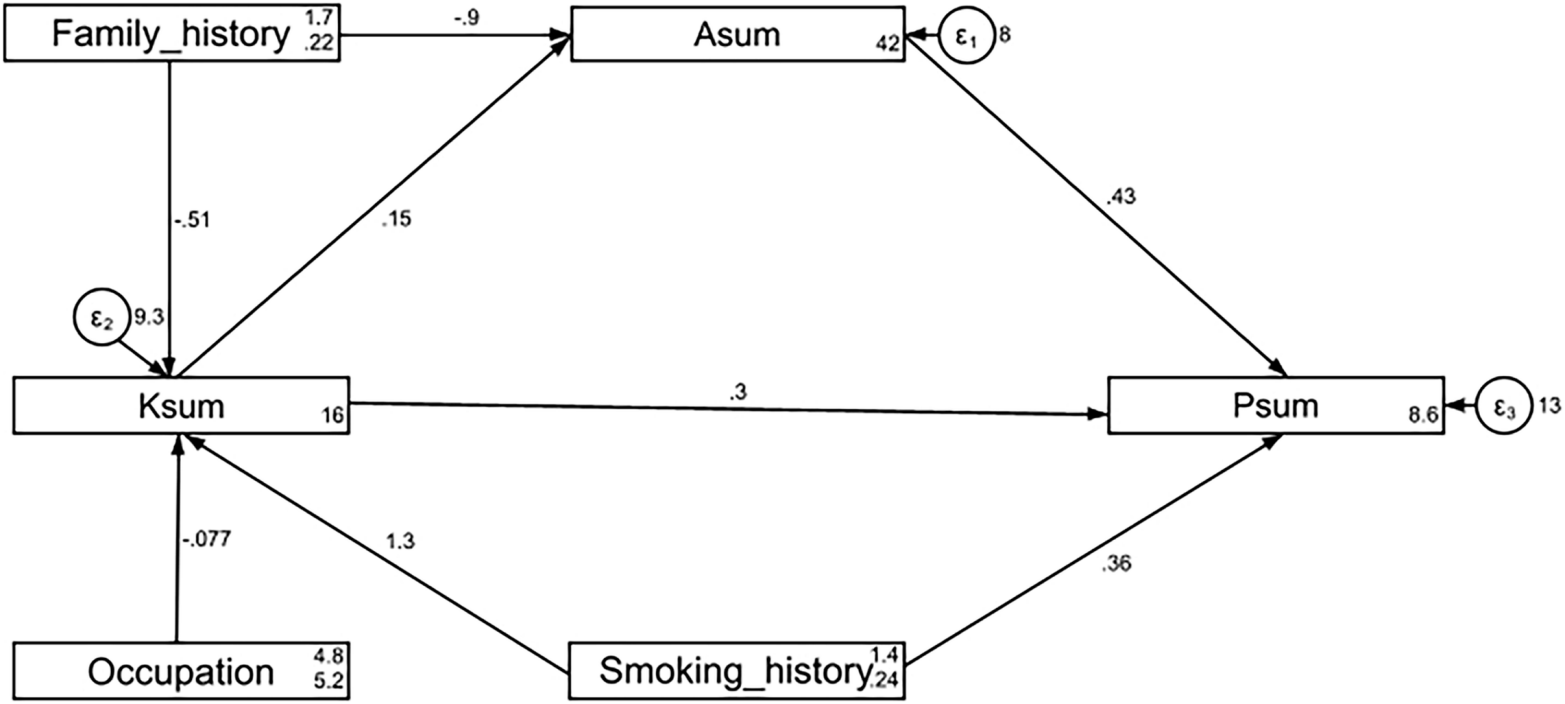
SEM model.

The fit indices of the SEM model reached the desired range, indicating good model fit results (RMSEA value: 0.000, SRMR value: 0.015, TLI value: 1.027, and CFI value: 1.000) (Supplementary Table S1), the pathway results showed that smoking history (*β* = 1.29, *p* < 0.001) directly affected knowledge. Family history (*β* = −0.90, *p* < 0.001) and knowledge (*β* = 0.15, *p* < 0.001) directly affected attitude. Knowledge (*β* = 0.43, *p* < 0.001) and attitude (*β* = 0.30, *p* < 0.001) directly affected practice (Supplementary Table S2 and Figure 1). Indirect effects analyses also showed that smoking history (*β* = 0.19, *p* = 0.002) had an indirect effect on attitude. Smoking history (*β* = 0.47, *p* < 0.001), family history (*β* = −0.57, *p* < 0.001), and knowledge (*β* = 0.06, *p* < 0.001) had indirect effects on practice (Supplementary Table S3).

## Discussion

Asthma patients in Inner Mongolia exhibit adequate knowledge, positive attitudes, and proactive practices in differentiating between allergic rhinitis accompanied by asthma and common coughs. Clinical programs should focus on enhancing patient education and training healthcare providers in rural areas to improve diagnostic accuracy and treatment effectiveness for respiratory conditions.

The findings of this study present a diverse spectrum of KAP among asthma patients in Inner Mongolia, specifically in their ability to differentiate between allergic rhinitis accompanied by asthma and common coughs. The results reveal adequate knowledge levels and attitudes, essential for effective asthma management. These findings align with other studies that emphasize the importance of patient education in chronic disease management and highlight that gaps in knowledge and practice can lead to suboptimal disease control and compliance (12, 13). However, while the study indicates a general adequacy in patient understanding and behavior, the variation in scores across different demographics suggests that specific groups may still experience gaps in knowledge and practice, potentially impacting disease control and patient compliance.

Significant differences in KAP scores were observed across educational levels and occupations, with lower scores generally seen in less educated groups and certain occupations like farmers and freelancers. This pattern is supported by the results from the multivariate logistic regression, which also identified these demographic factors as significant predictors of KAP outcomes. For instance, farmers and freelancers showed notably poorer knowledge scores, which could be attributed to less access to healthcare information and resources (14, 15). Moreover, having an immediate family member with asthma significantly improved knowledge and attitudes, a finding that underscores the role of personal experience and familial support in disease management (16, 17). This association was evident in both the significant differences analysis and multivariate logistic regression.

The relationships among the KAP variables were further elucidated through correlation analysis and SEM. Both analyses demonstrated significant positive correlations between knowledge and practice, as well as between attitude and practice, indicating that better-informed patients are more likely to engage in beneficial practices. The SEM results also highlighted direct and indirect pathways by which historical smoking affects knowledge and practice, emphasizing the complex interplay of behavioral and environmental factors in asthma management. These findings are consistent with literature indicating that comprehensive asthma education programs can significantly improve patient outcomes by enhancing both knowledge and practices (18, 19).

The distribution of knowledge responses among asthma patients in Inner Mongolia highlights areas of concern, particularly regarding less familiar topics such as the relationship between allergic rhinitis and asthma, and specific seasonal impacts on these conditions. For instance, a significant portion of respondents lacked clarity on the increased incidence of asthma in allergic rhinitis patients and the seasonal occurrence of allergic rhinitis symptoms. This is consistent with findings from other studies indicating gaps in public understanding about the linkages between different allergic conditions and their triggers (20, 21). To address these knowledge gaps, targeted educational programs could be developed, focusing on the epidemiology of asthma and allergic rhinitis, and delivered through community health talks, pamphlets, and digital media campaigns (22, 23). These interventions should particularly target rural areas and less educated populations, as these groups showed notably lower knowledge scores in the study.

Attitudinal responses reflect a generally positive outlook on the management of allergic rhinitis and asthma; however, there were notable deficiencies in the perceived ability to differentiate between these conditions and common coughs. Nearly half of the participants felt they did not have enough knowledge to make this distinction, which could lead to delays in seeking appropriate medical care or the use of incorrect treatments. To improve this, healthcare providers should consider implementing decision-support tools in clinical settings that help patients better understand their symptoms and possible conditions (24). Additionally, patient education sessions that specifically address the differences in symptoms and appropriate actions could be incorporated into routine care, especially for patients with a family history of asthma, as they are likely to be more receptive to learning about related conditions (25, 26).

The practice dimension results indicated variability in how patients manage their conditions. While some proactive measures like reducing exposure to allergens were frequently reported, others, such as using different medications for allergic rhinitis versus asthma, were less commonly practiced. This suggests a disconnect between knowledge and application, potentially due to unclear guidance on when and how to use specific treatments effectively. Comparative studies have shown that practical management improvements often require not just patient education but also regular follow-ups and personalized management plans (27). Practical interventions could include the development of mobile apps that track symptoms and medication usage, and alert patients when a consultation is advisable. Additionally, workshops that simulate scenario-based learning on managing allergic rhinitis and asthma could help patients apply their knowledge in real-life situations (28–30).

This study has several limitations that warrant consideration. First, the cross-sectional design limits the ability to infer causality between the observed knowledge, attitudes, and practices and the management outcomes of asthma and allergic rhinitis. Second, the reliance on self-reported data may introduce response bias, as participants could overestimate their understanding or compliance. Third, the study was confined to a single geographical region and one healthcare facility, which may restrict the generalizability of the findings to other settings or populations within Inner Mongolia or other regions.

## Conclusion

This study demonstrates that asthma patients in Inner Mongolia generally possess adequate knowledge, positive attitudes, and proactive practices regarding the differentiation between allergic rhinitis accompanied by asthma and common coughs. However, disparities remain across different demographic groups, particularly among those with lower education levels or working in rural or freelance settings. Our findings underscore the importance of targeted educational interventions and tailored clinical support to enhance asthma self-management and reduce misdiagnosis or delayed treatment.

## Future work

Future research should consider conducting longitudinal studies to evaluate changes in knowledge, attitudes, and practices over time, especially in response to targeted educational interventions. Additionally, qualitative interviews may offer deeper insights into the barriers faced by underserved populations, such as rural residents or the older adults, in recognizing and managing overlapping symptoms. Expanding the study to include multiple hospitals across different regions of Inner Mongolia or other provinces could improve the generalizability of the findings. Finally, integrating mobile-based health education tools may offer a scalable and accessible method to enhance asthma and allergic rhinitis management in remote or digitally underserved populations.

## Data Availability

The original contributions presented in the study are included in the article/Supplementary material, further inquiries can be directed to the corresponding authors.
