# Supplementary material for: Knowledge, attitudes, and practices of asthma patients in Inner Mongolia regarding differentiation between allergic rhinitis accompanied asthma and cough
Source: Front Public Health. 2025 Dec 15;13:1624520. doi: 10.3389/fpubh.2025.1624520 (PMC12745466; doi:10.3389/fpubh.2025.1624520)
Supplement: Supplementary file 1 [file Table_1.DOCX]

**Table S1. Structural equation modeling (SEM) model fit indices including RMSEA, SRMR, TLI, and CFI**

| Indicators | Reference | Results |
| --- | --- | --- |
| RMSEA | <0.08 Good | 0.000 |
| SRMR | <0.08 Good | 0.015 |
| TLI | >0.8 Good | 1.027 |
| CFI | >0.8 Good | 1.000 |

**Table S2. Standardized path coefficients from the structural equation model (SEM). Bolded P-values indicate statistically significant effects at P < 0.05. P-values equal to 0 reflect values less than 0.001 due to rounding.**

| Structural |  | Estimate | P>\|z\| |
| --- | --- | --- | --- |
| Asum |  |  |  |
|  | Ksum | 0.15 | **0** |
|  | Family history | -0.90 | **0** |
| Psum |  |  |  |
|  | Asum | 0.43 | **0** |
|  | Ksum | 0.30 | **0** |
|  | Smoking history | 0.36 | 0.268 |
| Ksum |  |  |  |
|  | Family history | -0.51 | 0.069 |
|  | occupation | -0.08 | 0.177 |
|  | Smoking history | 1.30 | **0** |

**Table S3. Analysis of direct and indirect effects among key variables in the SEM model. Bolded values indicate statistically significant paths (P < 0.05).**

| Model paths |  | Total effects | | Direct Effect | | Indirect effect | |
| --- | --- | --- | --- | --- | --- | --- | --- |
|  |  | β (95% CI) | P | β (95% CI) | P | β (95% CI) | P |
| Asum <- |  |  |  |  |  |  |  |
|  | Ksum | 0.15(0.07,0.23) | <0.001 | 0.15(0.07,0.23) | **<0.001** | —— | —— |
|  | Family history | -0.97(-1.48,-0.46) | <0.001 | -0.90(-1.40,-0.39) | **<0.001** | -0.07(-0.17,0.01) | 0.098 |
|  | occupation | -0.01(-0.03,0.00) | 0.201 | —— | —— | -0.01(-0.03,0.00) | 0.201 |
|  | Smoking history | 0.19(0.07,0.32) | 0.002 | —— | —— | 0.19(0.07,0.32) | **0.002** |
| Psum <- |  |  |  |  |  |  |  |
|  | Asum | 0.43(0.32,0.54) | <0.001 | 0.43(0.32,0.54) | **<0.001** | —— | —— |
|  | Ksum | 0.36(0.26,0.47) | <0.001 | 0.30(0.20,0.40) | **<0.001** | 0.06(0.03,0.10) | **<0.001** |
|  | Family history | -0.57(-0.89,-0.26) | <0.001 | —— | —— | -0.57(-0.89,-0.26) | **<0.001** |
|  | occupation | -0.02(-0.07,0.01) | 0.185 | —— | —— | -0.02(-0.07,0.01) | 0.185 |
|  | Smoking history | 0.83(0.18,1.49) | 0.012 | 0.36(-0.27,1.00) | 0.268 | 0.47(0.24,0.71) | **<0.001** |
| Ksum |  |  |  |  |  |  |  |
|  | Family history | -0.50(-1.04,0.03) | 0.069 | -0.50(-1.04,0.03) | 0.069 | —— | —— |
|  | occupation | -0.07(-0.18,0.03) | 0.177 | -0.07(-0.18,0.03) | 0.177 | —— | —— |
|  | Smoking history | 1.29(0.76,1.82) | <0.001 | 1.29(0.76,1.82) | **<0.001** | —— | —— |
